# Advanced abdominal imaging with dual energy CT is feasible without increasing radiation dose

**DOI:** 10.1186/1470-7330-15-S1-P1

**Published:** 2015-10-02

**Authors:** M Uhrig, D Simons, HP Schlemmer

**Affiliations:** 1 German Cancer Research Centre (DKFZ) Heidelberg, Department of Radiology, ImNeuenheimer Feld 280, D-69120 Heidelberg, Germany

## Aim

Dual energy CT (DECT) has already proven its potential in oncological imaging, e.g. for contrast media quantification, tissue characterisation and monitoring targeted therapies. Considering that oncological patients have repeated follow-up examinations, dose issues should not be neglected. Purpose of this study was to evaluate radiation dose of conventional single energy CT (SECT) versus DECT abdominal imaging in clinical routine.

## Methods

100 patients (62y (± 14)) had either SECT (44) or DECT (56) in clinical routine. Computed tomography dose index (CTDI_vol_), dose length product (DLP) and CTDI normalised to amount of contrast media (CTDIn) were reported. CTDIvol was transformed to patient specific dose estimate (SSDE). Image noise (SD) was recorded as the mean measurement of three ROIs placed in subcutaneous fat and was normalised to absorbed dose by . Statistical significance was tested with two-sided t test (α < 0.05).

## Results

There was no significant difference of the reported parameter between DECT and SECT: mean DECT- CTDI_vol_ was 14.2 mGy (±3.9), mean SECT-CTDI_vol_ 14.3 mGy (±4.5). Mean DECT-DLP was 680 mGycm (±220), mean SECT-DLP 665 mGycm (±231). Mean CTDIn was for both DECT and SECT 0.11 mGy/ml (±0.02). Mean DECT-SSDE was 15.7 mGy (±1.9), mean SECT-SSDE 16.1 mGy (±2.5). Mean DECT-SDn was 42.2 HU*√mGy (±13.9), mean SECT-SDn 47.8 HU*√mGy (±14.9).

## Conclusion

Advanced abdominal imaging with DECT is feasible without increasing radiation dose. This is of special interest in oncology, where targeted therapies demand more than simple size measurements. Functional information from dual energy CT will, without dose penalty, contribute to sophisticated oncological imaging.

